# Supplementary material for: POLCAM: instant molecular orientation microscopy for the life sciences
Source: Nat Methods. 2024 Oct 7;21(10):1873–83. doi: 10.1038/s41592-024-02382-8 (PMC11466833; doi:10.1038/s41592-024-02382-8)
Supplement: Supplementary file 2 — Reporting Summary [file 41592_2024_2382_MOESM2_ESM.pdf]

# nature portfolio

Corresponding author(s): Steven Lee

Last updated by author(s): May 27, 2024

# Reporting Summary

Nature Portfolio wishes to improve the reproducibility of the work that we publish. This form provides structure for consistency and transparency in reporting. For further information on Nature Portfolio policies, see our Editorial Policies and the Editorial Policy Checklist.

## Statistics

For all statistical analyses, confirm that the following items are present in the figure legend, table legend, main text, or Methods section.

| n/a | Confirmed | |
|---|---|---|
| ☐ | ☒ | The exact sample size (*n*) for each experimental group/condition, given as a discrete number and unit of measurement |
| ☐ | ☒ | A statement on whether measurements were taken from distinct samples or whether the same sample was measured repeatedly |
| ☒ | ☐ | The statistical test(s) used AND whether they are one- or two-sided<br>*Only common tests should be described solely by name; describe more complex techniques in the Methods section.* |
| ☒ | ☐ | A description of all covariates tested |
| ☒ | ☐ | A description of any assumptions or corrections, such as tests of normality and adjustment for multiple comparisons |
| ☐ | ☒ | A full description of the statistical parameters including central tendency (e.g. means) or other basic estimates (e.g. regression coefficient) AND variation (e.g. standard deviation) or associated estimates of uncertainty (e.g. confidence intervals) |
| ☒ | ☐ | For null hypothesis testing, the test statistic (e.g. *F*, *t*, *r*) with confidence intervals, effect sizes, degrees of freedom and *P* value noted<br>*Give P values as exact values whenever suitable.* |
| ☒ | ☐ | For Bayesian analysis, information on the choice of priors and Markov chain Monte Carlo settings |
| ☒ | ☐ | For hierarchical and complex designs, identification of the appropriate level for tests and full reporting of outcomes |
| ☒ | ☐ | Estimates of effect sizes (e.g. Cohen's *d*, Pearson's *r*), indicating how they were calculated |

*Our web collection on statistics for biologists contains articles on many of the points above.*

## Software and code

Policy information about availability of computer code

| | |
|---|---|
| Data collection | The open-source software Micro-Manager (version 2.0.0) was used for microscope control and data acquisition, and downloaded from https://micro-manager.org/Download_Micro-Manager_Latest_Release. A Micro-Manager device adapter for the polarization camera used in this work was included as part of the free image acquisition software ThorCam (v3.6.0, Thorlabs), which was downloaded from https://www.thorlabs.com/software_pages/ViewSoftwarePage.cfm?Code=ThorCam. |
| Data analysis | Basic image inspection and image cropping was performed in ImageJ (v1.53t). Single-molecule data was analyzed using custom MATLAB (version R2022a, Mathworks) application POLCAM-SR for which the source code and installer are available on GitHub at https://github.com/ezrabru/POLCAM-SR. Diffraction-limited, high-dimensional polarisation camera image processing and visualization was performed using a custom plugin napari-polcam for the open source software napari (v0.4.17), installed using the instructions at https://napari.org/stable/. The source code for the plugin is available at https://github.com/ezrabru/napari-polcam. |

For manuscripts utilizing custom algorithms or software that are central to the research but not yet described in published literature, software must be made available to editors and reviewers. We strongly encourage code deposition in a community repository (e.g. GitHub). See the Nature Portfolio guidelines for submitting code & software for further information.

## Data

Policy information about availability of data

All manuscripts must include a data availability statement. This statement should provide the following information, where applicable:

- Accession codes, unique identifiers, or web links for publicly available datasets
- A description of any restrictions on data availability
- For clinical datasets or third party data, please ensure that the statement adheres to our policy

The datasets generated as part of this study were uploaded to Zenodo: pixel-dependent camera calibration results (\url{https://doi.org/10.5281/zenodo.10578307}), single SYTOX Orange on a cover glass (\url{https://doi.org/10.5281/zenodo.10469322}), PAINT data of single Nile red dyes binding to lipid bilayer-coated silica microspheres (\url{https://doi.org/10.5281/zenodo.10469444}), TAB-PAINT data (\url{https://doi.org/10.5281/zenodo.10470795}), dSTORM phalloidin-AF488 (\url{https://doi.org/10.5281/zenodo.10470982}), dSTORM phalloidin-AF647 (\url{https://doi.org/10.5281/zenodo.10732697}) and T cells (\url{https://doi.org/10.5281/zenodo.10471496}).

## Human research participants

Policy information about studies involving human research participants and Sex and Gender in Research.

| | |
|---|---|
| Reporting on sex and gender | n.a. |
| Population characteristics | n.a. |
| Recruitment | n.a. |
| Ethics oversight | n.a. |

Note that full information on the approval of the study protocol must also be provided in the manuscript.

# Field-specific reporting

Please select the one below that is the best fit for your research. If you are not sure, read the appropriate sections before making your selection.

☒ Life sciences ☐ Behavioural & social sciences ☐ Ecological, evolutionary & environmental sciences

For a reference copy of the document with all sections, see nature.com/documents/nr-reporting-summary-flat.pdf

# Life sciences study design

All studies must disclose on these points even when the disclosure is negative.

| | |
|---|---|
| Sample size | All experiments used in this study have been performed at least 3 times, to assure results generated by the presented method are repeatable. |
| Data exclusions | Localisations with less than 500 detected photons were excluded from all single-molecule datasets in this study, to avoid biased orientation estimates (as explained in the manuscript). |
| Replication | Each experiment presented in this work was replicated successfully at least 3 times to assure repeatability of the results. Single dye on glass or in polymer was replicated successfully >10 times on different optical setups, days, sample regions and different dyes. PAINT imaging of lipid-coated glass microspheres was successfully repeated >3 times on separate days and sample regions. TAB-PAINT imaging of alpha-synuclein fibrils was successfully repeated >3 times on different optical setups, days and sample regions. dSTORM imaging of the actin network of HeLa cells was successfully repeated 3 times (3 cells per labelling method). Live T cell imaging was successfully repeated 3 times on separate days and different sample regions. |
| Randomization | Randomization is not relevant to this study. |
| Blinding | Blinding is not relevant to this study. |

# Reporting for specific materials, systems and methods

We require information from authors about some types of materials, experimental systems and methods used in many studies. Here, indicate whether each material, system or method listed is relevant to your study. If you are not sure if a list item applies to your research, read the appropriate section before selecting a response.

## Materials & experimental systems

| n/a | Involved in the study | |
|---|---|---|
| ☐ | ☒ | Antibodies |
| ☐ | ☒ | Eukaryotic cell lines |
| ☒ | ☐ | Palaeontology and archaeology |
| ☒ | ☐ | Animals and other organisms |
| ☒ | ☐ | Clinical data |
| ☒ | ☐ | Dual use research of concern |

## Methods

| n/a | Involved in the study | |
|---|---|---|
| ☒ | ☐ | ChIP-seq |
| ☒ | ☐ | Flow cytometry |
| ☒ | ☐ | MRI-based neuroimaging |

# Antibodies

Antibodies used: OKT3 antibody (provided by the Human Immunology Unit, WIMM, Oxford, UK)

Validation: Unknown

# Eukaryotic cell lines

Policy information about cell lines and Sex and Gender in Research

Cell line source(s): HeLa cells: derived from cells isolated from the cervix of a 31-year-old female with adenocarcinoma.
COS7 cells: derived from the CV-1 cell line (ATCC CCL-70) by transformation with an origin defective mutant of SV40 which codes for wild type T antigen. This is an African green monkey kidney fibroblast-like cell line.
Jurkat T cells: clone of the Jurkat-FHCRC cell line (ATCC TIB-152), established from the peripheral blood of a 14-year-old, male, acute T-cell leukemia patient.

Authentication: The cell lines used were not authenticated.

Mycoplasma contamination: HeLa cells and Jurkat T cells are periodically tested for mycoplasma contamination and tested negative. It is unknown whether COS7 cells were tested for mycoplasma contamination.

Commonly misidentified lines (See ICLAC register): n.a.
