# Supplementary figures and images for: POLCAM: instant molecular orientation microscopy for the life sciences
Source: Nat Methods. 2024 Oct 7;21(10):1873–83. doi: 10.1038/s41592-024-02382-8 (PMC11466833; doi:10.1038/s41592-024-02382-8)

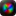

Supplement: Supplementary file 11 — MATLAB application for processing single-molecule polarization camera images (https://github.com/ezrabru/POLCAM-SR). [file 41592_2024_2382_MOESM11_ESM.zip › POLCAM-SR-main/POLCAM-SR/POLCAM-SR_resources/icon_16.png]

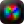

Supplement: Supplementary file 11 — MATLAB application for processing single-molecule polarization camera images (https://github.com/ezrabru/POLCAM-SR). [file 41592_2024_2382_MOESM11_ESM.zip › POLCAM-SR-main/POLCAM-SR/POLCAM-SR_resources/icon_24.png]

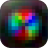

Supplement: Supplementary file 11 — MATLAB application for processing single-molecule polarization camera images (https://github.com/ezrabru/POLCAM-SR). [file 41592_2024_2382_MOESM11_ESM.zip › POLCAM-SR-main/POLCAM-SR/POLCAM-SR_resources/icon_48.png]

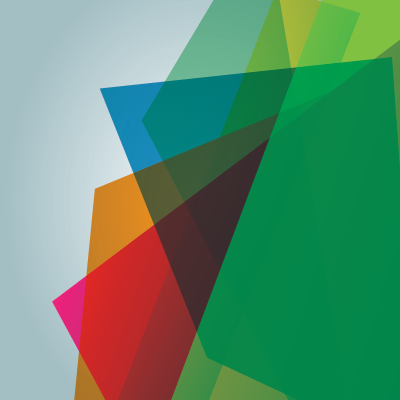

Supplement: Supplementary file 12 — MATLAB application for live processing of polarization camera images during acquisition (https://github.com/ezrabru/POLCAM-Live). [file 41592_2024_2382_MOESM12_ESM.zip › POLCAM-Live-main/POLCAM-Live/POLCAM_Live/for_redistribution_files_only/splash.png]

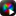

Supplement: Supplementary file 12 — MATLAB application for live processing of polarization camera images during acquisition (https://github.com/ezrabru/POLCAM-Live). [file 41592_2024_2382_MOESM12_ESM.zip › POLCAM-Live-main/POLCAM-Live/POLCAM_Live_resources/icon_16.png]

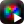

Supplement: Supplementary file 12 — MATLAB application for live processing of polarization camera images during acquisition (https://github.com/ezrabru/POLCAM-Live). [file 41592_2024_2382_MOESM12_ESM.zip › POLCAM-Live-main/POLCAM-Live/POLCAM_Live_resources/icon_24.png]

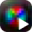

Supplement: Supplementary file 12 — MATLAB application for live processing of polarization camera images during acquisition (https://github.com/ezrabru/POLCAM-Live). [file 41592_2024_2382_MOESM12_ESM.zip › POLCAM-Live-main/POLCAM-Live/POLCAM_Live_resources/icon_32.png]

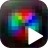

Supplement: Supplementary file 12 — MATLAB application for live processing of polarization camera images during acquisition (https://github.com/ezrabru/POLCAM-Live). [file 41592_2024_2382_MOESM12_ESM.zip › POLCAM-Live-main/POLCAM-Live/POLCAM_Live_resources/icon_48.png]

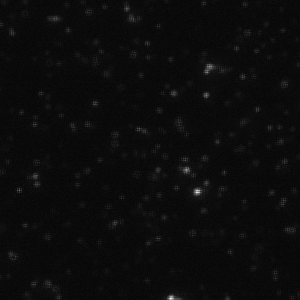

Supplement: Supplementary file 16 — SYTOX orange: representative unprocessed dataset of SYTOX orange dispersed on a cover glass. [file 41592_2024_2382_MOESM16_ESM.tif]

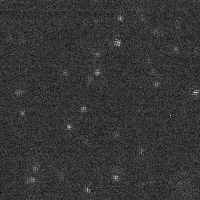

Supplement: Supplementary file 18 — dSTORM phalloidin-AF488: representative unprocessed dSTORM data of fixed HeLa cells labeled with phalloidin-AF488, showing highly linearly polarized emission (that is, rotationally restricted fluorophores). [file 41592_2024_2382_MOESM18_ESM.tif]

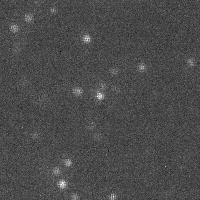

Supplement: Supplementary file 19 — dSTORM phalloidin-AF647: representative unprocessed dSTORM data of fixed HeLa cells labeled with phalloidin-AF647, showing less linearly polarized emission (that is, less rotationally restricted fluorophores). [file 41592_2024_2382_MOESM19_ESM.tif]
