# Supplementary material for: POLCAM: instant molecular orientation microscopy for the life sciences
Source: Nat Methods. 2024 Oct 7;21(10):1873–83. doi: 10.1038/s41592-024-02382-8 (PMC11466833; doi:10.1038/s41592-024-02382-8)
Supplement: Supplementary file 12 — MATLAB application for live processing of polarization camera images during acquisition (https://github.com/ezrabru/POLCAM-Live). [file 41592_2024_2382_MOESM12_ESM.zip › POLCAM-Live-main/POLCAM-Live/POLCAM_Live/PackagingLog.html]

```
mcc -o POLCAM_Live -W 'WinMain:POLCAM_Live,version=1.0' -T link:exe -d C:\Users\ezrab\Documents\GitHub\POLCAM-Live\POLCAM-Live\POLCAM_Live\for_testing -v C:\Users\ezrab\Documents\GitHub\POLCAM-Live\POLCAM-Live\POLCAM_Live.mlapp -r C:\Users\ezrab\Documents\GitHub\POLCAM-Live\POLCAM-Live\POLCAM_Live_resources\icon.ico 
Compiler version: 8.4 (R2022a)

Analyzing file dependencies. 

Parsing file "C:\Users\ezrab\Documents\GitHub\POLCAM-Live\POLCAM-Live\POLCAM_Live.mlapp"
	(referenced from command line).
Generating file "C:\Users\ezrab\Documents\GitHub\POLCAM-Live\POLCAM-Live\POLCAM_Live\for_testing\readme.txt".
Packaging...
Creating the bundle...
Creating the install agent URL file...
Web based installer created at C:\Users\ezrab\Documents\GitHub\POLCAM-Live\POLCAM-Live\POLCAM_Live\for_redistribution\MyAppInstaller_web.exe.
Packaging complete.
Elapsed packaging time was: 6 seconds.
```
